# Tinea corporis by *Nannizia gypsea*: delayed diagnosis due to unusual presentation^☆^^☆☆^

**DOI:** 10.1016/j.abd.2020.05.012

**Published:** 2020-11-20

**Authors:** Juliana de Jesus Soares, Nathalie Andrade Sousa, Luna Azulay-Abulafia, Rosane Orofino Costa

**Affiliations:** a General Dermatology Clinic, Hospital Universitário Pedro Ernesto, Rio de Janeiro, RJ, Brazil; b Faculty of Medicine, Universidade do Estado do Rio de Janeiro, Rio de Janeiro, RJ, Brazil

**Keywords:** Antifungal, Corticosteroids, Fungus, *Tinea corporis*

## Abstract

Fungal infections by dermatophytes can present with unusual clinical manifestations, which can cause diagnostic difficulties. The authors present the case of a patient with cutaneous infection by *Nanizzia gypsea*, initially treated erroneously with topical corticosteroids due to a wrong diagnosis. It was cured after antifungal treatment.

The authors describe the case of a 43-year-old male patient, resident in Jacarepaguá, Rio de Janeiro, Brazil who, at dermatological examination, presented a discreetly pruriginous erythematous plaque, showing a nummular aspect, with peripheral pustules and tonsured hair, located on the right forearm. A similar adjacent lesion was observed; the patient indicated that their onset occurred after he returned from a farm in the countryside area of Rio de Janeiro (Fig. 1). He underwent oral treatment with cephalexin and topically with corticosteroids, without improvement. As diagnostic hypotheses, sporotrichosis and pyoderma were suggested. The biopsy of a skin fragment stained with H &-E showed an enlarged hair follicle containing hyaline hyphae in its distal portion (Fig. 2), which are also evident in the silver impregnation by Grocott's method (Fig. 3), and PAS staining. There was also an inflammatory reaction in the superficial dermis, with a predominance of mononuclear cells. The culture for common germs was negative. *Nannizzia gypsea* was isolated from the skin fragment on Mycosel agar, confirming the diagnosis of tinea corporis. He underwent treatment with cyclopyroxolamine spray, which led to cure after 30 days (Fig. 4).

**Figure 1:**
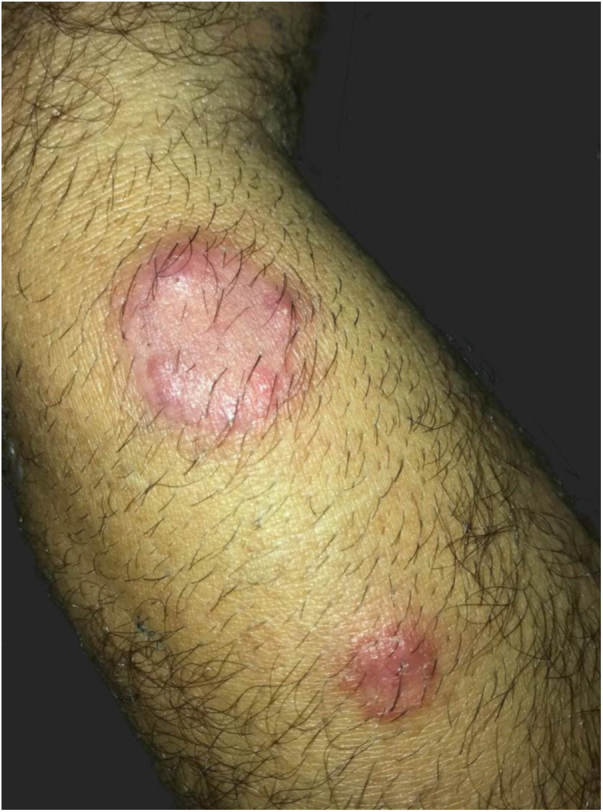
Clinical aspect of the lesions: nummular erythematous plaque, with peripheral pustules and tonsured hair, located on the right forearm, associated with a satellite lesion with the same characteristics.

**Figure 2:**
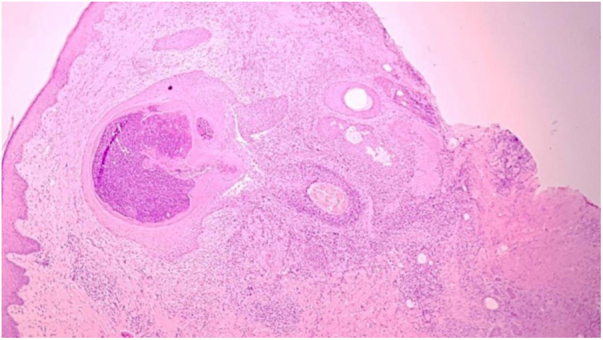
Histological section shows an enlarged hair follicle and inflammatory reaction in the superficial dermis, with a predominance of mononuclear cells (Hematoxylin & eosin, ×100).

**Figure 3:**
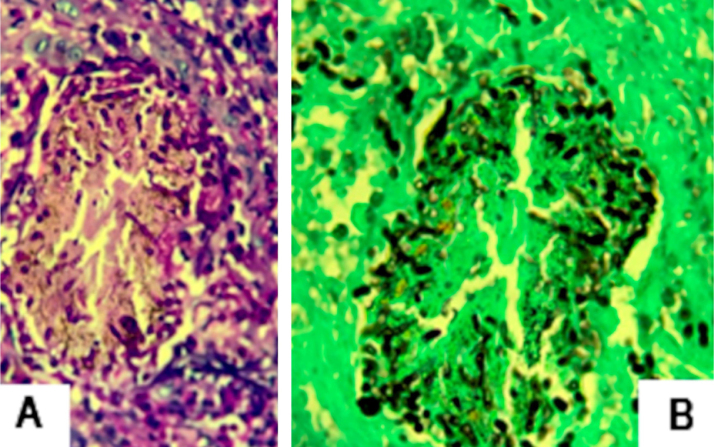
(A) Hyaline hyphae inside enlarged hair follicle (Hematoxylin & eosin, ×400). (B) Same, silver impregnation by the Grocott method, (Grocott, ×400).

**Figure 4:**
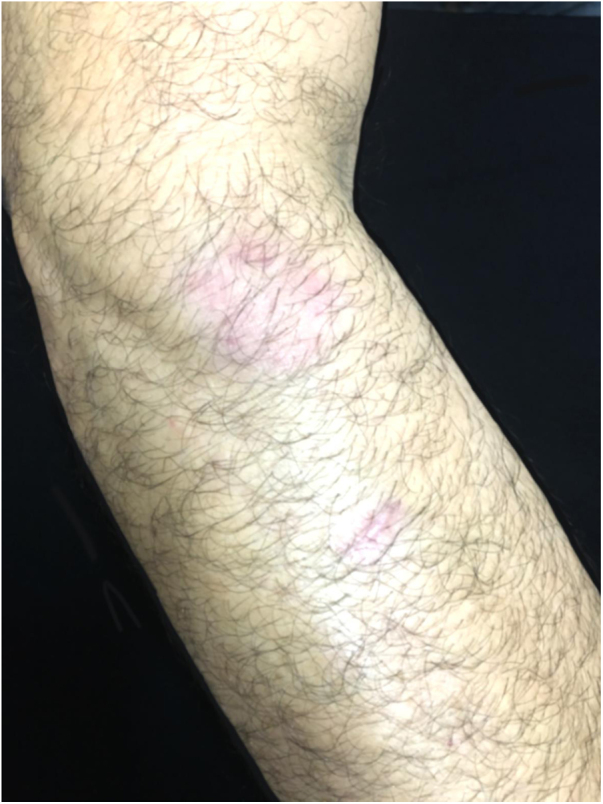
Clinical aspect of the lesions 30 days after treatment.

The prevalence of dermatophytoses caused by *Nannizia gypsea* ranges from 0.72% to 0.5%.^1^, ^2^ It is possible that the low prevalence is due to the exuberant inflammatory reaction, as this is a geophilic fungus, not adapted to human parasitism.^3^, ^4^ In general, the clinical presentation of tinea corporis is quite characteristic, presenting a mild or moderate inflammatory reaction, sometimes vesicular or vesicular-crusted, on the edge of a macular, erythematous squamous lesion.^5^ In this case, the clinical presentation of the lesion was not typical of dermatophytosis of the body; on the contrary, it was a plaque with pustules. In Rio de Janeiro, due to the hyperendemicity of sporotrichosis, this was, naturally, the first hypothesis. Currently, in the state of Rio de Janeiro, lesions that have an infiltrated aspect, without flaking, and that do not initially respond to systemic antibiotic therapy are suggestive of sporotrichosis.

Cases of dermatophytosis with atypical clinical presentation, as reported, may be mistakenly treated with ineffective or inadequate medications, if there are no specific complementary tests. Corticosteroids and antibiotics are the most common drugs used.^6^

In most cases, topical treatment with terbinafine, butenafine, cyclopyroxolamine, or azoles for two to four weeks is simple, cost-effective, and sufficient for healing.^7^, ^8^ Systemic treatment with oral antifungals, such as terbinafine and itraconazole, should be considered in immunosuppressed patients, in extensive and/or recurrent lesions, or in those who do not respond to the use of topical medications.

With the report of this case, the authors aimed to reinforce the idea that complementing the clinical diagnosis with specific laboratory tests that exclude or confirm the initial hypothetical clinical diagnoses can avoid unnecessary and costly treatments, which often prolong the suffering of patients.

## Financial support

None declared.

## Authors’ contributions

Juliana de Jesus Soares: Drafting and editing of the manuscript.

Nathalie Andrade Sousa: Drafting and editing of the manuscript.

Luna Azulay-Abulafia: Critical review of the manuscript.

Rosane Orofino Costa: Critical review of the manuscript.

## Conflicts of interest

None declared.
